# Evidence-Based Prevention of Preeclampsia: Commonly Asked Questions in Clinical Practice

**DOI:** 10.1155/2019/2675101

**Published:** 2019-08-01

**Authors:** Dagmar Wertaschnigg, Maya Reddy, Ben W. J. Mol, Fabricio da Silva Costa, Daniel L. Rolnik

**Affiliations:** ^1^Department of Obstetrics and Gynaecology, Monash University, Clayton, Victoria, Australia; ^2^Department of Obstetrics and Gynecology, Paracelsus Medical University, Salzburg, Austria; ^3^Monash Women's, Monash Health, Clayton Victoria, Australia; ^4^Department of Gynecology and Obstetrics, Ribeirão Preto Medical School, University of São Paulo, Ribeirão Preto, São Paulo, Brazil

## Abstract

In this review, we discuss the recent literature regarding the prevention of preeclampsia and aim to answer common questions that arise in the routine antenatal care of pregnant women. Prescription of low-dose aspirin for high-risk patients has been shown to reduce the risk of preeclampsia (PE). A daily dose between 100 and 150 mg taken in the evening should be initiated prior to 16 weeks of gestation and can be continued until delivery. Calcium supplementation seems to be advantageous but currently it is only considered for patients with poor dietary intake and high risk for PE. Recent data about heparin are still conflicting, and therefore, heparin can currently not be recommended in the prevention of PE.

## 1. Introduction

Low-dose aspirin as prevention of preeclampsia (PE) has been widely examined but indication for prescription, timing of treatment initiation, and dosage vary widely between the different studies and guidelines [1–6]. The most common reason to take aspirin has been a previous history of PE or any other high-risk factors according to maternal characteristics and medical history. Nevertheless, the detection rate for preeclampsia using history-based guidelines is at best 40% [7, 8]. A significant body of evidence now suggests that combined screening tests for PE outperform history-based screening and are now recommended by the Society of Ultrasound in Obstetrics and Gynecology (ISUOG) and the International Federation of Gynecology and Obstetrics (FIGO) [9–11]. The algorithm provided by the Fetal Medicine Foundation (FMF) combines maternal characteristics and history with the results of biophysical (mean arterial pressure (MAP), mean uterine artery pulsatility index (UtA-PI)) and biochemical measurements (serum placental growth factor (PLGF) and/or serum pregnancy-associated plasma protein-A (PAPP-A)) [12]. A patient-specific risk for the required delivery due to developed PE at a certain gestational age (GA) can be calculated, and detection rates (DR) range between 90% and 100% for PE <34 weeks and PE <32 weeks and 76.7% for preterm PE, but only 43.1% for term PE, at a 9.2% false positive rate [7, 12, 13].

This combined multimarker screening test is a valuable clinical tool as it provides a more specific indication for aspirin for a better-defined high-risk group [9, 14]. This article reviews the current evidence in the prevention of preeclampsia and answers common questions regarding how high-risk patients should be managed in clinical practice and the different preventative options available to reduce the incidence of hypertensive disorders in pregnancy.

### 1.1. Which Interventions Reduce the Risk of Preeclampsia?

#### 1.1.1. Aspirin

A large number of randomised controlled trials, systematic reviews, and meta-analyses have focused on the effectiveness of aspirin with varying doses and start times. This has resulted in conflicting conclusions, which can partly be explained by inconsistent reporting of outcome parameters, mixed subgroups, heterogeneous selection of high-risk women and treatment regimens, and differences in the gestational age at which treatment is initiated [4, 5]. The most recent meta-analysis showed that aspirin started before 16 weeks of gestation and at doses ≥100 mg/day at bedtime reduces the risk of preterm preeclampsia by approximately 70%, whereas an individual patient data meta-analysis reported a weaker reduction of 10% and a consistent effect throughout gestation [4, 5]. The weaker reduction in the latter meta-analysis could be explained by the fact that the majority of the included studies examined the use of aspirin at doses below 100 mg (21/23 trials) and started prophylaxis after 16 weeks of gestation (20/23 trials) [15]. The effect of aspirin in reducing the incidence of PE is likely to be a dose-, gestational age-, and adherence-dependent continuum.

The Aspirin for Evidence-Based Preeclampsia Prevention (ASPRE) study was a double-blind placebo-controlled randomised trial, which identified patients at high-risk of PE at 11-14 weeks of gestation using the FMF combined screening test, and then compared aspirin (150 mg per day at bed time) with placebo in those defined as high risk, from 11-14 weeks until 36 weeks of gestation. This landmark trial showed a significant reduction of 62% for preterm PE. There was no reduction on the incidence on term PE, but this may be due to a delay on the disease onset resulting in a shift of the distribution to the right [14].

#### 1.1.2. Heparin

Apart from antithrombotic therapy, low-molecular-weight heparin (LMWH) has the potential to redress the balance between pro- and antiangiogenic factors. Moreover, as heparin also has an anti-inflammatory action and does not cross the placenta, it has been considered a potential target for the prevention of placental mediated diseases [16]. However, conclusions about the use of LMWH alone or in combination with aspirin are conflicting [16, 17]. According to a recent systematic review and meta-analysis (eight trials, including 963 women), the use of LMWH showed a nonsignificant reduction in the risk of recurrence of placental mediated pregnancy complications of 36% (relative risk 0.64, 95% CI 0.36-1.11, p=0.11) [18]. The included multicentre trials could not prove a significant benefit of LMWH on prevention of PE, whereas single centre trials showed a beneficial effect. The latest meta-analysis from 2018 (seven trials with 1,035 women) about LMWH for secondary prevention showed a reduction in PE of 48% (RR 0.522 (95% CI: 0.334–0.815); p=0.004): in two of the three studies ranked as low quality there was a significant reduction of PE (89% and 73%, respectively); in the highly quality ranked trials, two of four had a significant reduction of PE (65% and 73%, respectively) [19]. Given this heterogeneity of data, currently no conclusion for clinical implementation of LWMH can be drawn. As a consequence, further large trials should be initiated to answer this question. For that reason, current guidelines endorsed by the American College of Obstetricians and Gynecologists (ACOG), the National Institute for Health and Care Excellence (NICE), and the Society of Obstetric Medicine of Australia and New Zealand (SOMANZ) do not recommend the use of LMWH in the prevention of PE [1–3]. Only the Society of Obstetricians and Gynaecologists of Canada (SOGC) discusses heparin as an option in women with a history of placental complications [20]. The indication of LMWH should be restricted to women with other comorbidities that require anticoagulation in pregnancy, such as antiphospholipid syndrome. However, a possible beneficial effect of combining low-dose aspirin and LMWH in preventing preeclampsia in this high-risk group is unclear [21].

#### 1.1.3. Calcium Supplementation

The evidence for general calcium supplementation for all women in prevention of hypertensive disorders is conflicting [22]. In a meta-analysis of 2014, a daily calcium supplementation of ≥ 1g in the second half of pregnancy showed a significant reduction of 55% for PE mainly for women under low intake diet (13 trials, 15,730 women: RR 0.45, 95% CI 0.31 to 0.65; I^2^ = 70%). There was a reduction of high blood pressure (12 trials, 15,470 women: risk ratio (RR) 0.65, 95% confidence interval (CI) 0.53 to 0.81; I^2^ = 74%) and a decrease in the composite outcome of maternal death or serious morbidity (four trials, 9732 women; RR 0.80, 95% CI 0.65 to 0.97; I^2^ = 0%) [23]. Whereas, a RCT from 2006 published by the WHO included 8325 women, calcium intake from 20 weeks of gestation did not prevent PE (4.1% vs. 4.5%) but only showed a significant reduction of severe hypertension (risk ratio, 0.71; 95% CI, 0.61-0.82) and maternal morbidity (risk ratio, 0.80; 95% CI, 0.70-0.91) [24]. Contrary to that, a recent placebo controlled RCT focused on the effect of calcium intake before and during the first half of pregnancy: preeclampsia occurred in 23% (69/296) in the calcium group versus 29% (82/283) in the placebo group (RR 0·80, 95% CI 0.61-1.06; p=0.121) [25]. However, in the subgroup of women with very good compliance of more than 80%, there was a significant reduction of PE in the calcium intake group (RR 0.66, CI 0.44-0.98; p=0.037). To conclude, currently a potential benefit of additional calcium intake (at doses of 1.0-2.0 g/day) is seen in women with a low calcium diet and especially for those at high risk of developing PE [1, 2, 20], but there is no evidence to support calcium supplementation in the general obstetric population.

#### 1.1.4. Folic Acid

Periconceptional supplementation with folic acid is necessary for normal placentation and reducing fetal risk for open neural tube defects [26]. Given only low evidence regarding reduction of PE, so far there has not been yet a clear recommendation to extend intake beyond 12 weeks of gestational age [27].

#### 1.1.5. Others

Oral antioxidants such as vitamins C and E, selenium, and coenzyme Q10 did not show any benefit regarding prevention of PE [28, 29]. Similarly, studies performed with fish oil, vitamins C and E, garlic, bed rest, salt restriction, progesterone, diuretics, and nitric oxide donors did not show a reduction of the PE risk [29–34], and therefore these interventions are not recommended by the obstetric societies guidelines.

### 1.2. What is the Mechanism by Which Aspirin Prevents Preeclampsia?

The exact pathogenesis of PE and the mechanism by which aspirin reduces its incidence remain unclear. Aspirin appears to be associated with a gestational age-dependant shift on the distribution of the disease to the right. The delay in the onset of preeclampsia seems greater at earlier gestational ages. This effect would then cause some cases of preterm PE to now occur at term, giving the impression that aspirin has little or no effect on PE at term. In patients with PE there is an imbalance of pro- and anti-inflammatory markers. In particular, there is an upregulation of Thromboxane A2 (TXA2), in relation to Prostacyclin (PGI2) [2, 6]. PGI2 and TXA2 are produced through the arachidonic acid pathway. Cyclooxygenase-1 and cyclooxygenase-2 (COX-1 and COX-2, respectively) act on arachidonic acid (AA) to produce Prostaglandin H2 (PGH2), which in turn leads to the formation of PGI2 and TXA2. PGI2, which is produced in endothelial cells, is an effective vasodilator and has anti-inflammatory properties. Conversely, TXA2 promotes platelet aggregation and is a potent vasoconstrictor. Different from other reversible-acting nonsteroid anti-inflammatory drugs (NSAID), acetyl-salicylic-acid (ASA, the active principle of aspirin) irreversibly blocks cyclooxygenase isoenzyme-1, especially at daily doses below 300 mg. As platelets are enucleated and have no nuclear DNA, they are unable to resynthesize COX-1, which leads to a decrease in Thromboxane A_2_ (TXA2) and weaker aggregation of platelets. Hence, it can be assumed that aspirin influences the imbalance of these prostaglandins by decreasing the vasoconstrictive action of TXA2.

Additionally,* in vitro* studies have shown that aspirin increases PlGF gene expression and production, as well as correcting imbalances in cytokines [35]. Hence, aspirin may also play a role in PE prevention through its anti-inflammatory action and endothelium stabilisation. It is also plausible that the regulation of cytokines provided by aspirin results in better trophoblastic invasion, which essentially occurs prior to 16-18 weeks of gestation [35].

### 1.3. When is Aspirin Indicated for the Prevention of Preeclampsia?

Using the combined screening algorithm by Fetal Medicine Foundation (FMF), the ASPRE trial chose a risk cut-off of 1:100 for preterm PE to define the high-risk group, which led to a detection rate (DR) of 77% for an 11% screen-positive rate (SPR) [14]. In 2013, ACOG recommended prescription of aspirin only either in patients with a history of early-onset PE and preterm delivery before 34 weeks of gestational age or for women with two or more previous pregnancies complicated by PE [2]. Compared to this very restricted recommendation in 2013, recently, ACOG expanded their indication to all patients with any high-risk factors and consider aspirin in case of several moderate-risk factors, which is roughly in agreement with the advice provided by NICE, SOGC, and SOMANZ (Table 1) [1–3, 6, 20, 36]. However, screening methods based on the use of maternal characteristics and history alone to identify the high-risk population, albeit simple to perform, detect only 40% of the cases of preeclampsia that will require delivery before 37 weeks of gestation [7].

### 1.4. Is Aspirin Safe for Use in Pregnancy?

Aspirin use during pregnancy seems safe for both mother and fetus. Treatment with aspirin did not show an increased risk for congenital malformations and did not have any negative effect on fetal development, nor on bleeding complications in the neonatal period [37–39]. Despite side effects such as minor vaginal bleeding and gastrointestinal symptoms, which occur in approximately 10% of patients, there is no evidence of increased risk of major maternal bleeding and no association with placental abruption [14]. Concerns regarding premature closure of the fetal arterial duct have never been confirmed. However, there is lack of data regarding possible side effects and long-term outcomes when prescribed in large scale to low-risk patients [14, 37, 38].

### 1.5. Aspirin: When to Start?

The majority of trials using aspirin to prevent placental complications have initiated treatment at or after 12 weeks of gestation [6]. There is now convincing evidence that the strongest reduction of preterm PE is achieved with commencement of therapy prior to 16 weeks of gestation [4]. However, incidence of PE may still be positively influenced when aspirin is initiated only after 16 weeks of gestation and, given its safety profile, high-risk women who present for antenatal care after 16 weeks of gestation may still benefit from prophylaxis. Important to note, this aspect has been discussed controversially debated in the literature and the maximum prophylactic effect seems to occur when started early [4, 5]. Moreover, when ingested at bedtime, aspirin decreases blood pressure and reduces the incidence of PE, preterm delivery, and fetal growth restriction [40].

### 1.6. What is the Ideal Dose of Aspirin and Until When?

The most commonly evaluated daily doses of aspirin vary between 60 and 162 mg. However, both* in vitro* and* in vivo* studies have demonstrated that the optimal dose is ≥ 100 mg/day [4, 35]. It also appears that there is a clear dose-dependent effect. In a study published by Caron et al., at a daily dosage of 81 mg, 121 mg, and 162 mg, 30%, 10%, and 5% of the individuals were classified as nonresponders, respectively [41]. Therefore, doses below 100 mg should be avoided [4], although direct comparisons of different dose regimens in randomised trials are not available.

### 1.7. Is There an Increased Risk of Bleeding Complications in Women Receiving Aspirin? And Should Patients Stop Taking Aspirin When Delivery Is Imminent?

Most randomised controlled trials (RCTs) and meta-analysis have not found a significant increase in major bleeding complications, and in absence of other anticoagulants, neuraxial blockade is not contraindicated [14, 15]. The ASPRE trial ceased aspirin at 36 weeks of gestation but treating until delivery is considered safe. There are no studies evaluating if ceasing prophylaxis at an earlier gestational age would have similar efficacy.

### 1.8. Is Aspirin Still Recommended to High-Risk Patients First-Seen after 16 Weeks of Gestation?

The most recent meta-analysis was unable to identify a statistically significant reduction of preterm PE in patients who initiated aspirin > 16 weeks of gestation, irrespective of whether the dose was above (relative risk 0.88, 95% CI 0.54; 1.43) or below 100 mg (relative risk 1.00, 95% CI 0.88; 1.25) [4]. Another meta-analysis did not find significant differences on the effect of aspirin according to the gestational age at which treatment is started, but this study did not analyse the combined effect of starting aspirin before 16 weeks and at doses higher than 100 mg [5]. ACOG still recommends initiation between 12 weeks and 28 weeks of gestation, but optimally before 16 weeks [6]. Considering that treatment with low-dose aspirin is cheap and appears safe, initiation after 16 weeks of gestation can still be recommended.

### 1.9. Should We Offer Aspirin to All Pregnant Women?

Prevention of PE with aspirin seems to be safe and inexpensive [36]. For these reasons, universal prophylaxis has been discussed [42]. However, aspirin prophylaxis for PE has predominately been evaluated in high-risk women, and it may not have the same effect in low-risk women [43]. In pregnancy, this is compounded by the general advice that it is beneficial to avoid unnecessary medication. Routine use of aspirin has been tested in low-risk women to assess acceptability with reported good adherence of 90%. However, half of the women approached declined randomisation because they did not want to take aspirin without a good reason [44]. Furthermore, rates of minor vaginal bleeding and postpartum haemorrhage (without influencing the need for blood transfusion) were higher in the aspirin group. In another prospective randomised multicentre study, daily aspirin at 100mg did not decrease the incidence of preeclampsia in low-risk nulliparous women when compared to placebo and adherence to treatment was of only 48.7% [43].

There is concern that if aspirin is prescribed universally without screening, it would likely reduce overall adherence rates. As a consequence, adherence in high-risk women could be weaker if they are not explicitly declared as high-risk patients [36]. Further RCTs are also required to assess safety and efficacy in low-risk populations [44].

### 1.10. Does Aspirin Prevent Other Adverse Pregnancy Outcomes?

Most randomised trials on prevention of PE with aspirin were underpowered to detect differences in other outcomes. Secondary analyses of the ASPRE trial suggest a 68% reduction in length of stay in the neonatal intensive care unit (NICU) mainly due to a reduction in early-onset PE and a reduction in the total number of small-for-gestational-age (SGA) infants [45–47]. In addition, previous meta-analyses could also show a significant benefit of aspirin regarding the risk of stillbirth, preterm birth, and SGA at birth [15, 47, 48].

### 1.11. Should We Offer Aspirin to Patients with Chronic Hypertension?

A secondary subgroup analysis of the ASPRE trial indicated that advice of aspirin for patients with chronic hypertension may not decrease the incidence of preterm PE in this specific group (adjusted odds ratio 1.29; 95%; CI 0.33-5.12) [49]. However, these results should be interpreted with caution as the trial was underpowered for subgroup analyses. Given aspirin is safe and further confirmation is still lacking, patients with chronic hypertension should still be offered aspirin.

### 1.12. What to Do with High-Risk Patients Who Report Known Allergy to Aspirin?

In patients with a known allergic reaction to aspirin (i.e., urticaria) or other contraindications (bleeding disorders, severe asthma, etc.), aspirin should not be used. High-risk patients in general and in particular those who cannot take aspirin should be followed up closely. As mentioned before, high-risk patients who cannot take aspirin may benefit from LMWH or calcium supplementation in specific cases, and these interventions should be considered on an individual case basis following adequate counselling and evaluation of risks and benefits.

### 1.13. How Should We Follow up High-Risk Women?

Women at high risk for PE should be closely monitored for signs of the disease, as well as the development of small-for-gestational-age (SGA) babies. During the second half of pregnancy in case of symptoms of PE, the implementation and use of sFLT-1/PlGF ratio with its excellent negative predictive value of 99.3% (95% CI, 97.9 - 99.9%) can help to exclude PE within the following week [50]. Patients presenting with markedly increased blood pressure might benefit from antihypertensive therapy to avoid transition into severe hypertension, which could be shown to be associated with adverse maternal and perinatal outcome [51]. Prescription of low-dose aspirin leads to a significant reduction in the number of SGA fetuses as well as stillbirths and preterm births [15, 46, 48]. For the detection of SGA pregnancies, serial assessment of fetal growth in the third trimester would be initiated [36].

## 2. Conclusion

Treatment with aspirin at a minimal daily dose of 100 mg (ideally 150 mg) taken at bedtime and initiated before 16 weeks of gestation reduces the incidence of PE. The ideal screening method is the use of maternal characteristics and history associated with biomarkers to calculate the individual risk. Aspirin is considered safe and has tolerable side effects but given the lack of data regarding adherence and long-term morbidity, primary prevention with universal prescription is not recommended. The additional benefit of LMWH still needs further investigation. Calcium supplementation is beneficial in women with a low calcium diet and especially for those at high risk of developing PE. Further trials with focus on prevention of late-onset PE as well as new therapeutic options for PE are underway.

## Figures and Tables

**Table 1:** Indication for aspirin and risk factors according to SOMANZ, NICE, USPSTF, and ACOG. Table 1 is reproduced from Wertaschnigg et al. (2019) [36] [under the Creative Commons Attribution License/public domain].

SOMANZ-RANZOG	NICE 2010	USPSTF 2014	ACOG 2018
*Indication for Aspirin:*	*Indication for Aspirin:*	*Indication for Aspirin:*	*Indication for Aspirin:*

*2 moderate or 1 high risk factor*	*2 moderate or 1 high risk factor*	*1 high risk factor*	*1 high risk factor*
*Dose*: unclear	*Dose*: 75 mg/d from 12 weeks	*Dose*: 81 mg/d optimally before 16 weeks	*Dose*: 81 mg/d optimally before 16 weeks
Until 37 weeks or until delivery	Continue daily until delivery.	Continue daily until delivery.	Continue daily until delivery.
	*Consider Aspirin:*	*Consider Aspirin:*	
		If more than one moderate risk factor	Other established medical indications
			

(i) *Risk factors*	*High risk factors*	*High risk factors*	*High risk factors*

(ii) Previous pregnancy with PE	(i) Previous pregnancy with PE	(i) Previous pregnancy with PE	(i) Previous pregnancy with PE

(iii) chronic hypertension	(ii) Chronic hypertension	(ii) Chronic hypertension	(ii) Chronic hypertension

(iv) Autoimmune disease	(iii) Autoimmune disease	(iii) Systemic lupus erythematosus	(iii) Systemic lupus erythematosus

(v) Diabetes mellitus	(iv) Diabetes mellitus	(iv) Diabetes mellitus	(iv) Diabetes mellitus

(vi) Chronic kidney disease	(v) Chronic kidney disease	(v) Chronic kidney disease	(v) Chronic kidney disease

(vii) Multifetal gestation		(vi) Multifetal gestation	(vi) Multifetal gestation

(viii) Nulliparity		(vii) Thrombophilia	(vii) Thrombophilia

(ix) Age >40 years	*Moderate risk factors*	*Moderate risk factors*	*Moderate risk factors*

(x) Inter-pregnancy interval >10 years	(i) Nulliparity	(i) Nulliparity	(i) Nulliparity

(xi) BMI at first visit >35 kg/m^2^	(ii) Age >40 years	(ii) Age >35 years	(ii) Age >35 years

(xii) Family history of PE	(iii) Inter-pregnancy interval >10 years	(iii) Inter-pregnancy interval >10 years	(iii) Inter-pregnancy interval >10 years

(xiii) Conception by IVF	(iv) BMI at first visit >35 kg/m^2^	(iv) BMI >30 kg/m2	(iv) BMI >30 kg/m2

(xiv) any risk factor	(v) Family history of PE	(v) Family history of PE	(v) Family history of PE

		(vi) History of SGA or adverse outcome	(vi) History of SGA or adverse outcome

		(vii) Sociodemographic characteristics (African American race or low socioeconomic status)	(vii) Sociodemographic characteristics (African American race or low socioeconomic status)

BMI: body mass index; IVF: *in vitro* fertilisation; PE: preeclampsia; SGA: small-for-gestational-age;
